# Subcellular location prediction of apoptosis proteins using two novel feature extraction methods based on evolutionary information and LDA

**DOI:** 10.1186/s12859-020-3539-1

**Published:** 2020-05-24

**Authors:** Lei Du, Qingfang Meng, Yuehui Chen, Peng Wu

**Affiliations:** 1 School of Information Science and Engineering, University of Jinan, Jinan, 250022 China; 2 Shandong Provincial Key laboratory of Network Based Intelligent Computing, Jinan, 250022 China

**Keywords:** Subcellular location, Position-specific scoring matrix, Consensus sequence, Absolute entropy correlation analysis, Linear discriminant analysis

## Abstract

**Background:**

Apoptosis, also called programmed cell death, refers to the spontaneous and orderly death of cells controlled by genes in order to maintain a stable internal environment. Identifying the subcellular location of apoptosis proteins is very helpful in understanding the mechanism of apoptosis and designing drugs. Therefore, the subcellular localization of apoptosis proteins has attracted increased attention in computational biology. Effective feature extraction methods play a critical role in predicting the subcellular location of proteins.

**Results:**

In this paper, we proposed two novel feature extraction methods based on evolutionary information. One of the features obtained the evolutionary information via the transition matrix of the consensus sequence (CTM). And the other utilized the evolutionary information from PSSM based on absolute entropy correlation analysis (AECA-PSSM). After fusing the two kinds of features, linear discriminant analysis (LDA) was used to reduce the dimension of the proposed features. Finally, the support vector machine (SVM) was adopted to predict the protein subcellular locations. The proposed CTM-AECA-PSSM-LDA subcellular location prediction method was evaluated using the CL317 dataset and ZW225 dataset. By jackknife test, the overall accuracy was 99.7% (CL317) and 95.6% (ZW225) respectively.

**Conclusions:**

The experimental results show that the proposed method which is hopefully to be a complementary tool for the existing methods of subcellular localization, can effectively extract more abundant features of protein sequence and is feasible in predicting the subcellular location of apoptosis proteins.

## Background

Apoptosis, also known as programmed cell death, is a basic biological phenomenon that is associated with the occurrence of a wide variety of diseases, such as a tumor, autoimmune disease, Alzheimer’s disease and so on. It plays an important role in animal development and homeostasis [1]. Studies have shown that apoptosis proteins are essential in this process. The subcellular location of a protein is closely related to its function. And only in the specific subcellular location, can the protein work [2]. Subcellular localization information of apoptosis proteins contributes to exploring the internal mechanism of programmed cell death and designing new drugs [3]. However, the number of apoptosis proteins with clear subcellular location markers is limited in the database, and it is time-consuming and costly to label samples by traditional experimental methods. Therefore, exploring feasible computational methods to predict the subcellular location of the given protein has been a hotspot for nearly two decades.

Over the years, various computational methods have been proposed in apoptosis protein subcellular localization. In 2006, Zhang et al. [4] built an apoptosis protein dataset with 225 proteins in total (ZW225). They proposed using a grouped weight of protein sequence and support vector machine (SVM) to predict the subcellular location of apoptosis proteins (EBGW_SVM). The overall accuracy achieved 83.1% by jackknife test. Chen et al. [5] constructed a different apoptosis protein dataset in 2007, which contains 317 proteins (CL317). By using the increment of diversity (ID), the highest jackknife predictive result was 82.7%. In the same year, they proposed another method called ID_SVM which combined ID with SVM to predict the subcellular location of apoptosis proteins [6]. The ID_SVM algorithm achieved a higher prediction accuracy by jackknife test, on CL317 dataset is 84.2% and ZW225 dataset is 85.8%. Zhang et al. [7] applied the concept of distance frequency and SVM to obtain the highest overall accuracy of 88.0% and 84.0% on CL317 dataset and ZW225 dataset, respectively. Liu et al. [8] extracted the evolutionary information embedded in the position-specific scoring matrix (PSSM) and combined it with auto covariance transformation to establish a PSSM-AC model. The overall accuracy achieved 91.5% on CL317 dataset and 84.0% on ZW225 dataset. Nearly, a series of advances have been achieved in the prediction of apoptosis protein subcellular location [9, 10]. Liang et al. [11] fused two feature descriptors named the frequency of triplet codons in the RNA sequence (FTC) and detrended forward moving-average cross-correlation analysis (DFMCA) to predict the subcellular location of apoptosis proteins, which reached the overall accuracy of 89.0% and 85.3% on CL317 dataset and ZW225 dataset, respectively. Li et al. [12] proposed two feature extraction methods namely generalized chaos game representation (GCGR) and novel statistics and information theory (NSI), they also combined them with other features including PseAAC and dipeptide composition. The jackknife prediction accuracy of CL317 dataset and ZW225 dataset was 92.7% and 87.1%, respectively by using SVM.

In summary, to identify the subcellular location of proteins, diverse methods have been proposed which mainly focus on the following two aspects: feature extraction and classification techniques. Feature extraction methods of protein sequences are the key to the prediction of subcellular location. Existing methods include protein amino acid composition (AAC) [13–15], pseudo-amino acid composition (PseAAC) [16, 17], physicochemical properties [18, 19], position-specific scoring matrix (PSSM) [20], Gene Ontology (GO) [21, 22] and so on. As for the classifier, numerous classifiers have been applied to solve the problem of protein subcellular localization, such as support vector machine (SVM) [23, 24], KNN [25, 26] and neural network [15, 27]. Among them, SVM is used extensively for its good classification performance and fast computing speed.

Some early studies used GO information as the feature to solve this problem and achieved the most significant improvement. However, for new proteins, because of the lack of GO information, it is hard to use the GO terms. Recently, evolutionary information based features extracted from position-specific scoring matrix (PSSM) have shown their effectiveness in subcellular localization [19]. Xie et al. [28] proposed a model named LOCSVMPSI which utilized the PSSM and four-part amino acid compositions as the feature vector. Huang et al. [29] formulated a protein sequence with the pseudo position-specific scoring matrix (PsePSSM). Dehzangi et al. [30] proposed a feature extraction method named PSSM-S to predict the subcellular location of Gram-positive and Gram-negative proteins. Wan et al. [31] combined the profile-alignment features and PseAA features to predict the localization of chloroplast proteins. Liang et al. [32] constructed a PSSM-based model by using Geary autocorrelation function and DCCA coefficient for apoptosis protein subcellular localization prediction. Xiang et al. [33] utilized the proportion of the golden section to split PSSM and proposed segmented evolutionary information to represent protein sequences. Wang et al. [34] proposed segmented amino acid composition in PSSM (PSSM-SAA) to tackle the subcellular localization problem. All these methods have shown that based on evolutionary information, discriminative features can be extracted for classification. Therefore, using PSSM to extract effective features to represent protein sequences is still an outstanding problem.

To solve the problem of insufficient information in a single feature set, researchers have paid attention to fuse multiple features to formulate protein sequences in recent years. Zhang et al. [35] fused Moran autocorrelation and cross correlation with PSSM to get protein sequence information, then the principal component analysis was used to reduce redundant and irrelevant information. Wan et al. [36] adopted a linear neighborhood propagation (LNP) classifier ensemble scheme to incorporate both split amino-acid composition (SAAC) features and profile-alignment (PA) features for predicting subchloroplast localization. Qu et al. [37] presented a method to predict the subcellular location of multi-site proteins by combining N-terminal signals, pseudo amino acid composition, physicochemical property, stereo-chemical property and amino acid index distribution. However, the fusion feature vectors usually established by splicing multiple different features and the features integrated through this method often have a high dimension [11, 38–40]. The high-dimensional features contain a good deal of redundant information which may have a harmful influence on performing the classifier. Dimensionality reduction algorithms can help to eliminate the redundant data from the original feature space and are widely used in machine learning [41].

In this paper, we focus on extracting features based on evolutionary information embedded in position-specific scoring matrix (PSSM). Two novel feature extraction methods are therefore proposed to predict the subcellular locations of apoptosis proteins. First, according to PSSM, we transform the protein primary sequence into the consensus sequence, and propose a novel feature extraction method based on the consensus sequence, which named consensus sequence-based transition matrix (CTM). The CTM feature reflects distributions information of the amino acid transitions. For each protein sequence, CTM method can obtain a 40-dimensional feature vector. Then we propose another feature extraction method calculated from PSSM matrix directly. It can establish a 190-dimensional feature named absolute entropy correlation analysis (AECA-PSSM). AECA-PSSM derives from relative entropy or KL divergence, and it reflects the relationship between each two columns of PSSM. Thus, for a given protein sequence, we can generate a 230-dimensional fusion feature. Next linear discriminant analysis (LDA) is used to eliminate the noise and reduce the dimension of the proposed features. Finally, the feature vector after dimensionality reduction is fed into SVM to identify the subcellular location. And the proposed CTM-AECA-PSSM-LDA method reaches a higher classification performance in identifying subcellular locations of apoptosis proteins on CL317 and ZW225 datasets.

## Results

### Performance of the two proposed feature extraction methods

In this paper, the protein samples are formulated with two evolutionary information based feature extraction methods: consensus sequence-based transition matrix (CTM) and absolute entropy correction analysis (AECA-PSSM). To investigate the effectiveness of the proposed method, we first test the performance of the two novel feature extraction methods.

**Fig. 1:**
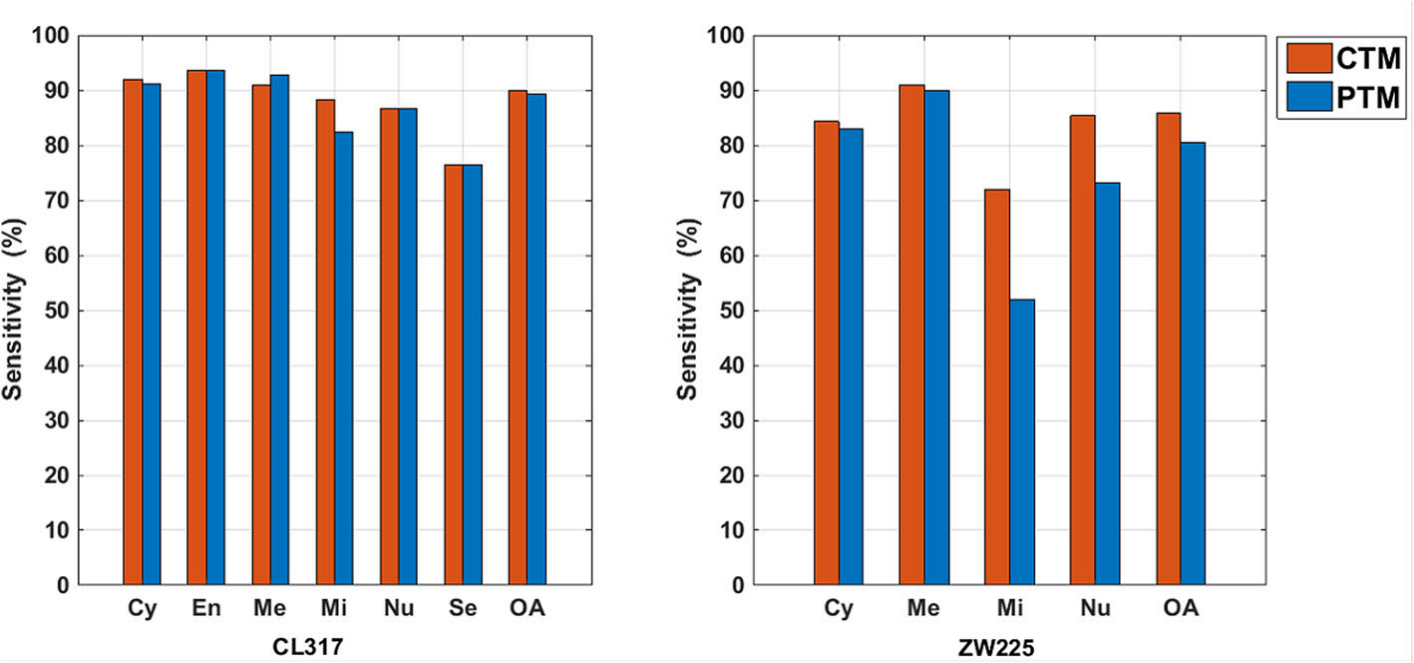
Comparison between primary sequence-based transition matrix (PTM) feature and consensus sequence-based transition matrix (CTM) feature for the two datasets

In the consensus sequence-based transition matrix method, we utilize the consensus sequence transformed from the protein primary sequence to integrate evolutionary information embedded in PSSM. Then, for the consensus sequence enriched with evolutionary information, we construct a transition matrix to extract features. Figure 1 shows the comparison between features extracted from the transition matrix of protein primary sequence (PTM) and the transition matrix of consensus sequence (CTM). On the whole, the CTM method performs better than PTM method. That’s because compared with the protein primary sequence, the consensus sequence obtained by PSSM contains the evolutionary information of the protein. Therefore, the proposed consensus sequence-based transition matrix method provides more discriminating information than the protein primary sequence.

Tables 1 and 2 show the classification results of CTM feature extraction method on CL317 and ZW225, respectively. In Table 1, we find that on CL317 dataset, the overall accuracy is 89.91% and the sensitivity of secreted proteins is lower than other five subcellular locations. As can be seen from Table 2, on ZW225 dataset, the overall accuracy obtained by CTM method achieves 85.78% and expect mitochondrion proteins, the sensitivity of other subcellular locations is 84.29%-91.01%.

**Table 1:** Classification results of CTM feature for the CL317 dataset

location	Jackknife test					
	Cy	En	Me	Mi	Nu	Se
Sen(%)	91.96	93.62	90.91	88.24	86.54	76.47
Spe(%)	91.71	100	98.47	98.94	96.98	100
Acc(%)	91.80	99.05	97.16	97.79	95.27	98.74
MCC	0.82	0.96	0.90	0.88	0.83	0.87
F	0.89	0.97	0.92	0.90	0.86	0.87
OA(%)	89.91

**Table 2:** Classification results of CTM feature for the ZW225 dataset

location	Jackknife test			
	Cy	Me	Mi	Nu
Sen(%)	84.29	91.01	72.00	85.37
Spe(%)	92.90	92.65	99.00	95.11
Acc(%)	90.22	92.00	96.00	93.33
MCC	0.77	0.83	0.78	0.78
F	0.84	0.90	0.80	0.82
OA(%)	85.78			

For the absolute entropy correlation analysis method, we use a novel analytical method derived from KL divergence to measure the relationship between each two columns in PSSM. Tables 3 and 4 show the classification results of AECA-PSSM feature extraction method on CL317 dataset and ZW225 dataset, respectively. It can be seen from Table 3 that on CL317 dataset, except the secreted proteins, the sensitivity in other subcellular locations is 84.62%-94.55% and the overall accuracy is 89.91%. From Table 4, we can find that on ZW225 dataset, the sensitivity of mitochondrion proteins is lower than other subcellular locations and the overall accuracy of the entire dataset reaches of 85.78%.

**Table 3:** Classification results of AECA-PSSM feature for the CL317 dataset

location	Jackknife test					
	Cy	En	Me	Mi	Nu	Se
Sen(%)	91.07	91.49	94.55	91.18	84.62	76.47
Spe(%)	95.12	99.63	96.95	98.59	96.98	99.67
Acc(%)	93.69	98.42	96.53	97.79	94.95	98.42
MCC	0.86	0.94	0.88	0.89	0.82	0.83
F	0.91	0.95	0.90	0.90	0.85	0.84
OA(%)	89.91

**Table 4:** Classification results of AECA-PSSM feature for the ZW225 dataset

location	Jackknife test			
	Cy	Me	Mi	Nu
Sen(%)	87.14	94.38	68.00	75.61
Spe(%)	92.90	90.44	98.00	97.83
Acc(%)	91.11	92.00	94.67	93.78
MCC	0.79	0.84	0.71	0.78
F	0.86	0.90	0.74	0.82
OA(%)	85.78			

The sequence similarity has a significant influence for the prediction performance and the lower the sequence similarity is, the more difficult the prediction is. According to the above experimental results, we can find that the classification results of the two proposed feature extraction methods are lower in ZW225 dataset. The possible reason is that the ZW225 dataset has lower sequence similarity than the CL317 dataset, but the results are acceptable. The secreted proteins in CL317 dataset and mitochondrion proteins in ZW225 dataset have the lower sensitivity. The reason may be that the sample sizes of these two subcellular locations in the datasets are small and the classifier tends to predict samples as majority classes.

### Effect of different feature extraction methods

One of the most important but also most difficult problems in computational biology is to convert the protein sequence into an effective numerical representation, which is known as feature extraction. In this paper, two novel evolutionary information based feature extraction methods are proposed to represent protein sequence information. After getting the CTM feature and AECA-PSSM feature, we combined them to form a 230 dimensional fusion feature vector: CTM-AECA-PSSM. However, as more sequence information is obtained by combining the two features, it also brings more noise, which has a negative impact on the predictor. Then we project the 230-dimensional fusion feature into a *p*=*C*−1 dimensional feature space by LDA dimensionality reduction method. Table 5 shows the contributions of different feature extraction methods on CL317 dataset and ZW225 dataset.

**Table 5:** Classification results of different feature extraction methods

Dataset	Feature extraction method	Jackknife test (%)						
		Sensitivity						
		Cy	En	Me	Mi	Nu	Se	OA
CL317	CTM	91.96	93.62	90.91	88.24	86.54	76.47	89.91
	AECA-PSSM	91.07	91.49	94.55	91.18	84.62	76.47	89.91
	CTM-AECA-PSSM	92.86	91.49	92.73	85.29	88.46	76.47	90.22
	CTM-AECA-PSSM-LDA	99.11	100	100	100	100	100	99.68
ZW225	CTM	84.29	\	90.01	72.00	85.37	\	85.78
	AECA-PSSM	87.14	\	94.38	68.00	75.61	\	85.78
	CTM-AECA-PSSM	87.14	\	88.76	76.00	80.49	\	85.33
	CTM-AECA-PSSM-LDA	97.14	\	91.01	100	100	\	95.56

**Fig. 2:**
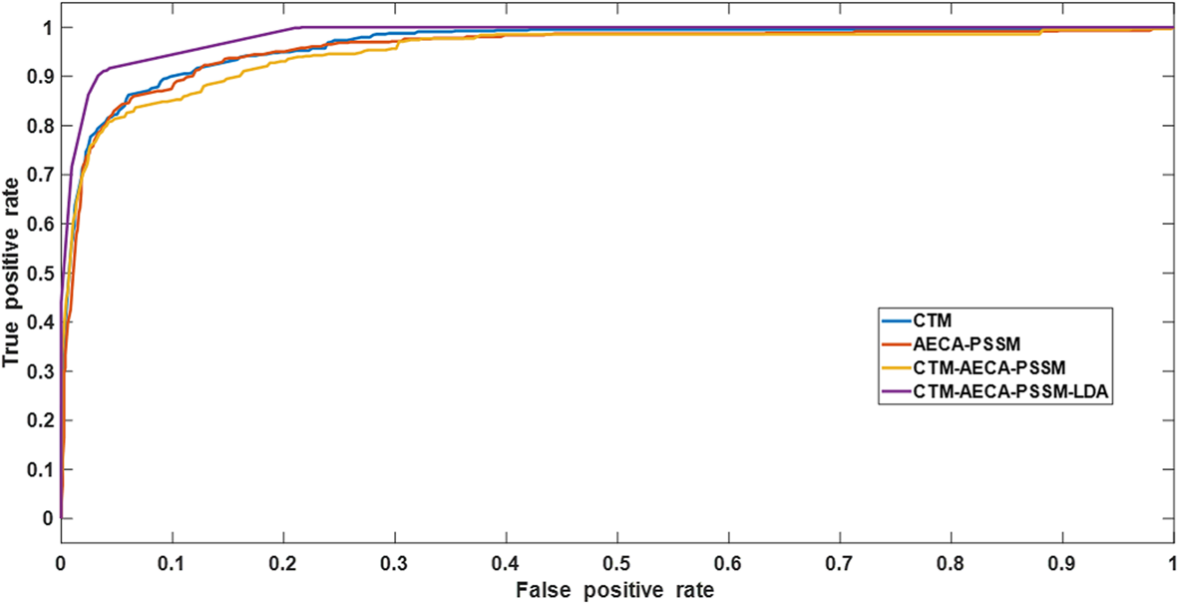
The ROC curves of different feature extraction methods on CL317 dataset

**Fig. 3:**
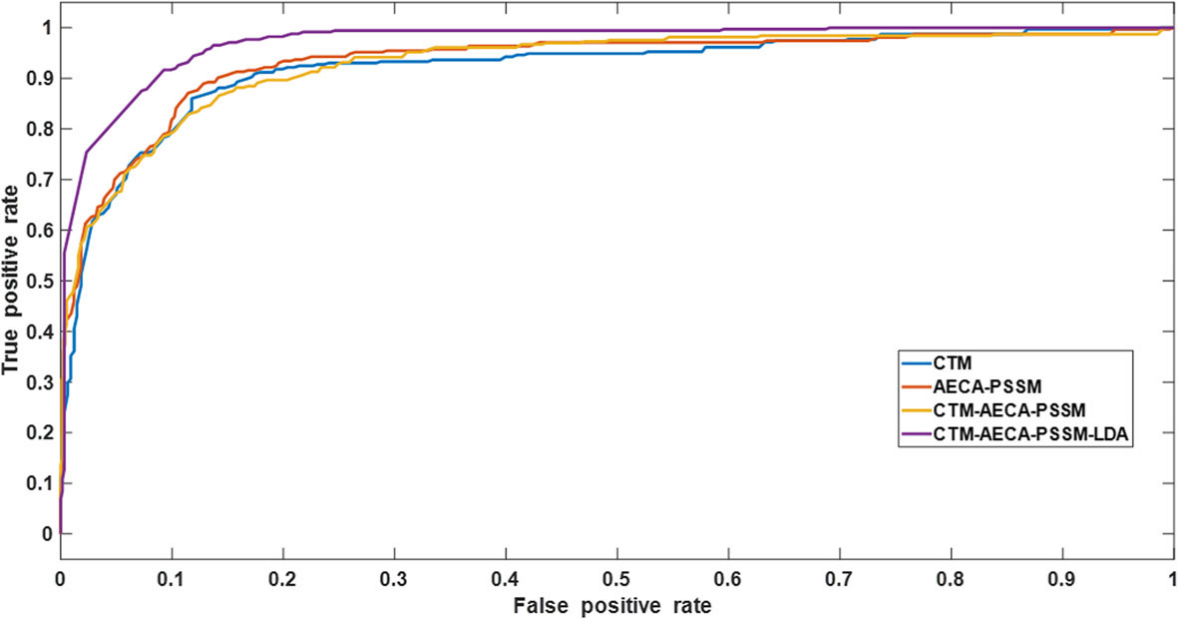
The ROC curves of different feature extraction methods on ZW225 dataset

We listed in Table 5 the sensitivity of each subcellular location and the overall accuracy of different feature extraction methods in CL317 and ZW225 datasets. On CL317 dataset, we can get a better prediction results which reach the overall accuracy of 90.22% by using CTM-AECA-PSSM method. And on ZW225 dataset, the overall accuracy after fusing the CTM algorithm and AECA-PSSM algorithm is 85.33%, which is 0.45% lower than using the two algorithms alone. The reason may be that when the two features are fused by generating a higher-dimensional feature vector, we can only get more information from the protein sequence, but the noise caused by the redundant and irrelevant information can not be eliminated which may make the performance of the classifier worse. When the two features are fused, the role of them is not fully played. Therefore, the prediction accuracy of CTM-AECA-PSSM on ZW225 dataset decreased slightly. From Table 5, we can also find that after dimensionality reduction using LDA, the prediction results are significantly improved on both CL317 dataset and ZW225 dataset. It indicates that LDA can effectively eliminate the redundant and irrelevant information and improve the accuracy of subcellular localization. To further assess the robustness of the model using different feature extraction methods, Figs. 2 and 3 show the ROC curves using the four different feature extraction algorithms on CL317 dataset and ZW225 dataset, respectively.

### Prediction results of different classification algorithms

In this paper, we consider four different classification algorithms, including extreme learning machine (ELM) [42], K-nearest neighbors (KNN) [34], logistic regression (LR) [43] and support vector machine (SVM). The prediction results under the four classifiers by jackknife test on the CL317 dataset and ZW225 dataset are shown in Table 6. It can be seen from Table 6 that the four classifiers have ideal prediction results on the two datasets which shows the effectiveness of our extracted features. On CL317 dataset, all of these four classifiers achieve the overall accuracy more than 99%. SVM, ELM and LR all achieve the highest overall accuracy of 99.68%. On ZW225 dataset, the overall accuracy achieved by the four classifiers are more than 93% and SVM achieves the highest overall accuracy 95.56%. Therefore, in this paper, we choose SVM as the final classification prediction algorithm.

**Table 6:** Prediction results of different classifiers

Dataset	classifier	Jackknife test (%)						
		Sensitivity						
		Cy	En	Me	Mi	Nu	Se	OA
CL317	ELM	99.11	100	100	100	100	100	99.68
	KNN	99.11	100	98.18	100	100	100	99.37
	LR	99.11	100	100	100	100	100	99.68
	SVM	99.11	100	100	100	100	100	99.68
ZW225	ELM	91.43	\	92.13	92.00	100	\	93.33
	KNN	91.43	\	91.01	100	100	\	93.78
	LR	92.86	\	92.13	100	100	\	94.67
	SVM	97.14	\	91.01	100	100	\	95.56

### Classification results of the proposed method

Prediction of apoptosis protein subcellular localization is an important research content in bioinformatics. In this work, we propose a method named CTM-AECA-PSSM-LDA to identify the subcellular location of apoptosis proteins. First the two proposed feature extraction methods CTM and AECA-PSSM are employed to represent the protein sequence. Then dimensionality reduction is performed by LDA. Finally, the sample data after dimensionality reduction are classified by SVM. The results of jackknife test on CL317 and ZW225 datasets are presented in Table 7. From Table 7, we can see that the OA for CL317 dataset and ZW225 dataset by our method achieve 99.68% and 95.56%, respectively. The experimental results indicate that the method can effectively predict the subcellular location of apoptosis proteins.

**Table 7:** Classification results of the proposed CTM-AECA-PSSM-LDA method

Locations	Jackknife test									
	CL317					ZW225				
	Sen (%)	Spe (%)	Acc (%)	MCC	F	Sen (%)	Spe (%)	Acc (%)	MCC	F
Cy	99.11	100	99.68	0.99	1	97.14	94.84	95.56	0.90	0.93
En	100	100	100	1	1	\	\	\	\	\
Me	100	99.62	99.68	0.99	0.99	91.01	98.53	95.56	0.91	0.94
Mi	100	100	100	1	1	100	100	100	1	1
Nu	100	100	100	1	1	100	100	100	1	1
Se	100	100	100	1	1	\	\	\	\	\
OA (%)	99.68					95.56				

### Comparison with the other prediction methods

In this section, to further evaluate the effectiveness of the proposed method, we compare it with some previous methods on the same apoptosis protein datasets. Tables 8 and 9 show the prediction results of different methods on CL317 dataset and ZW225 dataset, respectively. All the results are obtained using jackknife test. The OA of the two datasets and the sensitivity of each subcellular class are listed.

**Table 8:** Comparison from different methods on CL317 dataset by jackknife test

Methods	Jackknife test (%)						
	Sensitivity						OA
	Cy	En	Me	Mi	Nu	Se	
ID [5]	81.3	83.0	81.8	85.3	82.7	88.2	82.7
ID_SVM [6]	91.1	87.2	89.1	79.4	73.1	58.8	84.2
DF_SVM [7]	92.9	86.5	85.5	76.5	93.6	76.5	88.0
PSSM-AC [8]	93.8	95.7	90.9	91.2	86.5	82.4	91.5
Liang et al. [32]	92.9	93.6	89.1	82.4	84.6	76.5	89.0
Zhang et al. [44]	96.1	100	95.7	93.9	95.5	98.0	96.0
FTC-DFMCA-PSSM [11]	92.9	93.6	89.1	82.4	86.5	93.6	89.0
MACC-PSSM [35]	96.4	93.6	94.5	82.4	80.8	76.5	90.5
Chen et al. [10]	95.5	94.1	93.6	96.4	94.2	94.1	94.8
ERT-ECT-PSSM-IS [45]	93.8	94.1	100	97.9	96.2	92.7	95.0
CTM-AECA-PSSM-LDA	99.1	100	100	100	100	100	99.7

**Table 9:** Comparison from different methods on ZW225 dataset by jackknife test

Methods	Jackknife test (%)				
	Sensitivity				OA
	Cy	Me	Mi	Nu	
EBGW_SVM [4]	90.0	93.3	60.0	63.4	83.1
ID_SVM [6]	92.9	91.0	68.0	73.2	85.8
DF_SVM [7]	87.1	92.1	64.0	73.2	84.0
PSSM-AC [8]	82.9	92.1	68.0	78.0	84.0
Liang et al. [32]	87.1	89.1	68.0	75.6	84.4
Zhang et al. [44]	93.5	92.1	96.0	93.5	92.2
MACC-PSSM [35]	88.6	92.1	64.0	75.6	84.9
FTC-DFMCA-PSSM [11]	88.6	93.3	64.0	75.6	85.3
ERT-ECT-PSSM-IS [45]	80.0	91.0	92.0	87.8	87.1
CTM-AECA-PSSM-LDA	97.1	91.0	100	100	95.6

Based on CL317 dataset, the performance of the proposed CTM-AECA-PSSM-LDA model is compared with ten previous predictors. The OA of these methods range from 82.7% to 99.7%, among which CTM-AECA-PSSM-LDA achieves the highest prediction accuracy (99.7%). The sensitivity of endoplasm proteins, membrane proteins, mitochondrion proteins, nucleus proteins and secreted proteins achieve 100% in our method. And for the cytoplasm proteins, the sensitivity reaches of 99.1% which is also the highest.

Similarly, based on ZW225 dataset, the proposed CTM-AECA-PSSM-LDA prediction model is compared with nine other existing methods. The OA of our method (95.6%) is higher than other predictors test in this study. The sensitivity of mitochondrion proteins and nucleus proteins (both 100%) are the highest among all the methods which shows the excellent ability of our method in identifying mitochondrion and nucleus proteins. Moreover, the highest sensitivity of cytoplasm proteins (97.1%) also achieves by our method.

## Discussion

Apoptosis is a kind of elementary life phenomenon that exists widely in the biological world and apoptosis proteins play a significant role in this process. The function of apoptosis proteins is strongly related to their subcellular localization information. Facing the explosive growth of protein sequences in the post-genome era, to timely obtain useful information of sequences for drug design, it is an urgent need to develop computational methods for predicting the subcellular location of apoptosis proteins.

In this work, we propose a novel method to predict the subcellular location of apoptosis proteins named CTM-AECA-PSSM-LDA. Two novel feature extraction methods based on evolutionary information embedded in PSSM are designed. Firstly, the consensus sequence-based transition matrix (CTM) feature is extracted to reflect the amino acid transition information, and secondly, absolute entropy correlation analysis (AECA-PSSM) is proposed to obtain the relationship between each two columns of PSSM. After the two features mentioned above are fused together, LDA is adopted to reduce the dimension of the feature vector and eliminate the redundant information. Finally, SVM is regarded as a classifier to predict the subcellular location of apoptosis proteins. The overall accuracy by jackknife test is 99.7% and 95.6% for CL317 dataset and ZW225 dataset, respectively. Compared with other existing methods test in this paper, the overall accuracy is 3.7%-17% and 3.4%-12.5% higher on CL317 dataset and ZW225 dataset, respectively. The proposed CTM-AECA-PSSM-LDA method not only generates more discriminative features but also obtains satisfactory predictive performance on CL317 and ZW225 datasets, showing its potential for predicting apoptosis protein subcellular locations.

However, though our proposed CTM-AECA-PSSM-LDA method can effectively raise the prediction accuracy in two widely used benchmark datasets ZW225 and CL317, there are some disadvantages. Our method is trained on the two commonly used dataset and mainly consider the situation of single-site proteins that are ubiquitous in the existing apoptotic protein database. A series of recent publications [46–48] in demonstrating new findings or approaches pointed out that some proteins can simultaneously exist in or move between multiple subcellular locations. And these multi-site proteins usually have special functions and are of great search value. Given that it is difficult to collect a large enough multi-site apoptosis protein benchmark dataset meaningfully in statistics, similar to those of CL317 and ZW225 at present, which are the most widely used in previous studies, therefore, our method is still verified on those two datasets. In our future research, we will consider proteins with both single- and multi-site.

## Conclusion

The purpose of studying the subcellular location of apoptosis proteins is to further explore the intrinsic mechanism of programmed cell death and better understand the nature of life. Base on the evolutionary information, we propose two novel feature extraction methods to generate sequence feature of proteins. Then linear discriminant analysis algorithm is used to reduce the dimension of the extracted features. Finally SVM classifier is employed to predict the subcellular location of proteins. By jackknife test, on the two benchmark datasets CL317 and ZW225, the OA reach 99.7% and 95.6%, respectively. Experimental results show that the proposed method outperforms the previous predictors listed in the literature for most subcellular classes and indicate that it is promising for the recognition of subcellular locations. In general, our method is a relatively effective way to predict the subcellular location of apoptosis proteins. We hope that our method will be used as a complementary tool in the field of subcellular localization for proteins. According to a series of recent publications [49, 50], user-friendly and publicly accessible web-servers make great importance in building predictive system. We will make great efforts to provide a web-server for the proposed method in our future work.

## Methods

### Dataset

In this study, two benchmark datasets CL317 and ZW225 are applied to test the performance of the proposed method whose purpose is to determine the subcellular location of apoptosis proteins. The CL317 dataset was constructed in 2007 by Chen and Li [5]. It contains 317 protein sequences which located in six different subcellular locations respectively called cytoplasm (Cy), endoplasm (En), membrane (Me), mitochondrion (Mi), nucleus (Nu) and secreted (Se). The ZW225 dataset was established in 2006 by Zhang and Wang et al. [4]. It contains 225 protein sequences which located in four subcellular locations called cytoplasm (Cy), membrane (Me), mitochondrion (Mi) and nucleus (Nu). All of the two datasets are extracted from the SWISS-PROT database. Despite the small size of the two datasets, they are commonly used in the previous investigations [45, 51]. The details of two datasets CL317 and ZW225 are shown in Table 10.

**Table 10:** Details of the two datasets CL317 and ZW225

Dataset	Order	Subcellular localization	Number of proteins
CL317	1	Cytoplasm	112
	2	Endoplasm	47
	3	Membrane	55
	4	Mitochondrion	34
	5	Nucleus	52
	6	Secreted	17
ZW225	1	Cytoplasm	70
	2	Membrane	89
	3	Mitochondrion	25
	4	Nucleus	41

### The proposed feature extraction method

Effective feature extraction methods play a critical role in the subcellular location of proteins. In this paper, we propose two novel evolutionary information based feature extraction methods to effectively elucidate protein sequences. One of the feature extraction methods gets evolutionary information via the transition matrix of the consensus sequence (CTM). Another feature extraction method directly utilizes the evolutionary information from PSSM based on absolute entropy correlation analysis (AECA-PSSM).

To obtain the PSSM, PSI-BLAST program [52] is used to deal with the protein primary sequence from CL317 and ZW225 datasets. In our research, the non-redundant (NR) database is utilized, in the meantime, the E-value and the iterations numbers are respectively set to 0.001 and 3 [11]. The PSSM of a protein sequence with the length of *L* can be expressed by:

1 $$\begin{array}{@{}rcl@{}} PSSM=\left[\begin{array}{llll} {N_{1\rightarrow 1}}&{N_{1\rightarrow 2}}&{\cdots}&{N_{1\rightarrow 20}}\\ {N_{2\rightarrow 1}}&{N_{2\rightarrow 2}}&{\cdots}&{N_{2\rightarrow 20}}\\ {\vdots}&{\vdots}&{\vdots}&{\vdots}\\ {N_{i\rightarrow 1}}&{N_{i\rightarrow 2}}&{\cdots}&{N_{i\rightarrow 20}}\\ {\vdots}&{\vdots}&{\vdots}&{\vdots}\\ {N_{L\rightarrow 1}}&{N_{L\rightarrow 2}}&{\cdots}&{N_{L\rightarrow 20}} \end{array}\right] \end{array} $$

where *L* is the number of the amino acid residues in the protein sequence and *N*_*i*→*j*_ indicates the relative probability describing how the *i*th amino acid position in the protein sequence mutates into the *j* amino acid type during biological evolution processes. After we obtain the PSSM for a given protein, elements *N*_*i*→*j*_ in PSSM can be normalized by Eq. (2).

2 $$\begin{array}{@{}rcl@{}} P_{ij}=\frac{1}{1+e^{-N_{i \rightarrow j}}}  \end{array} $$

#### The proposed consensus sequence-based transition matrix (CTM) feature extraction method

Unlike many methods that extract features from the protein primary sequences, to integrate the evolutionary information, we attempt to extract features from the consensus sequences. After getting the PSSM of a given protein sequence *S*, the consensus sequence *S*^*c*^ enriched with evolutionary information can be obtained using the following formula:

3 $$\begin{array}{@{}rcl@{}} index_{i}=\arg\max(P_{i,j}:1\leq j \leq 20),1 \leq i \leq L  \end{array} $$

Through Eq. (3), we calculate the argument of the amino acid type corresponding to the maximum substitution probability in each row of the PSSM. Then, we can replace the *i*th amino acid residue located in the original protein sequence by the *index*_*i*_th amino acid type to obtain the consensus sequence. Through this process, we transform the protein primary sequence into a consensus sequence and integrate the evolutionary information.

In order to provide information about the distributions of the 20 amino acids transitions in the protein consensus sequences, we propose a feature extraction method based on the transition matrix of the consensus sequence.

For the consensus sequence $S^{c}=\left \{S_{1}^{c},S_{2}^{c},\cdots,S_{L}^{c}\right \}$ of a given protein sequence, we represent it as a directed graph *G*=(*V*,*E*). *V*=*v*_1_,*v*_2_,⋯,*v*_*n*_ is the set of vertices corresponding to the 20 types of amino acids, and *E*=*e*_1_,*e*_2_,⋯,*e*_*m*_ is the set of edges which model the pairwise relationship between amino acids. Since there are 20 types of amino acids that make up protein sequences, we can obtain 20×20 different combinations of amino acid pairs, which means that there are *m*=20×20 edges appear in graph *G*. In this paper, we use the occurrence number of the amino acid pairs to describe the pairwise relationship.

**Fig. 4:**
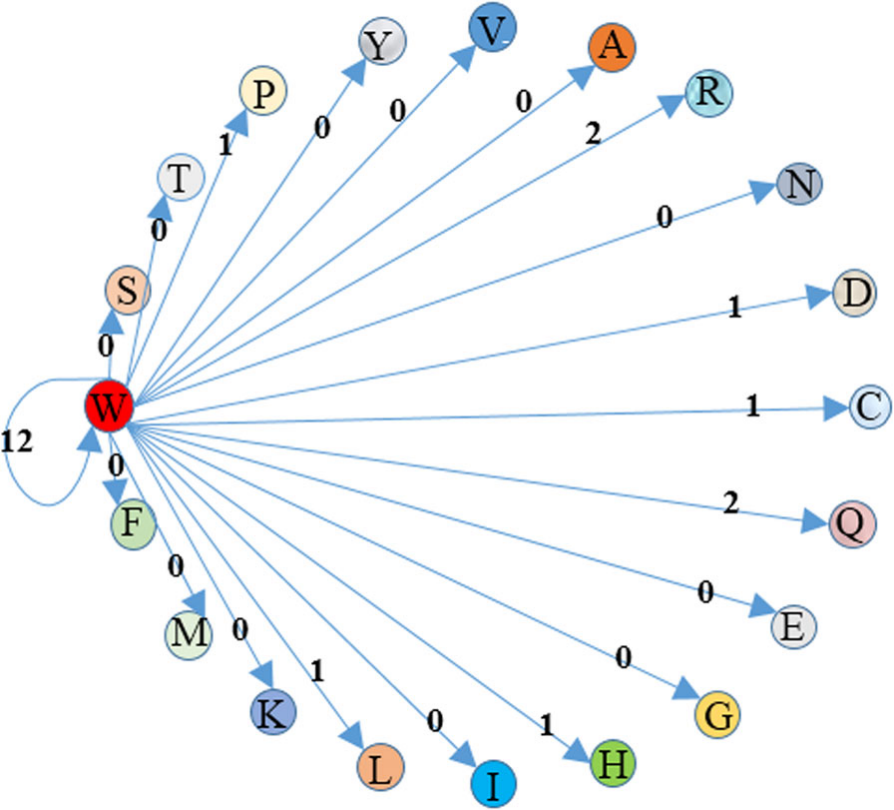
A sample example of the constructive process of graph *G*

In Fig. 4, we take a short consensus sequence as an example to demonstrate the construction of graph *G*. For a consensus sequence “CWWRCWWWLWWWRWQWWWWPWWCWDCWWWHCWWQ”, we only show the edges starting from node W (amino acid W)in graph *G*. In the consensus sequence, the occurrence of amino acid pairs “WW” is 12, so that the weight of the self-joining edge of node W is 12. And amino acid pairs “WC” occurs once in the sequence, so the edge starting from W to C weights 1.

The graph *G* can be represented as a transition matrix *T*(*G*)=*T*_*i*,*j*_,(*i*≤20,*j*≤20) which is denoted as:

4 $$\begin{array}{@{}rcl@{}} T(G)=\left[\begin{array}{llll} {T_{1,1}}&{T_{1,2}}&{\cdots}&{T_{1,20}}\\ {\vdots}&{\vdots}&{\vdots}&{\vdots}\\ {T_{i,1}}&{T_{i,2}}&{\cdots}&{T_{i,20}}\\ {\vdots}&{\vdots}&{\vdots}&{\vdots}\\ {T_{20,1}}&{T_{20,2}}&{\cdots}&{T_{20,20}} \end{array}\right] \end{array} $$

where *T*_*i*,*j*_ represents the nature of the weighted edges and we specify it as the occurrence number of corresponding amino acid pairs in the consensus sequence. The detail of the feature based on the transition matrix is as follows.

Firstly, we count the number of edges starting from each vertex so that we get the first feature descriptor based on the transition matrix of the consensus sequence. It can be obtained by:

5 $$\begin{array}{@{}rcl@{}} CTM_{i}^{1}=\sum_{j=1}^{20}T_{i,j},(i=1,\cdots,20)  \end{array} $$

The normalized value of *T*_*i*,*j*_ can be calculated by $p_{i,j}=\frac {T_{i,j}}{\sum _{r=1}^{20}\sum _{c=1}^{20}T_{r,c}} $. Then by applying the Shannon entropy to the normalized value of *T*_*i*,*j*_, we can obtain the second feature descriptor based on the transition matrix of consensus sequence. It reflects another attribute of each vertex and is obtained as follows:

6 $$\begin{array}{@{}rcl@{}} CTM_{i}^{2}=-\sum_{j=1}^{20}p_{i,j}log(p_{i,j}),(i=1,\cdots,20)  \end{array} $$

Finally, by using Eqs. (5) and (6), a 40-dimensional feature vector is established based on the transition matrix of the consensus sequence. The consensus sequence-based transition matrix (CTM) feature extraction method gets the distributions of the 20 amino acids transitions, rather than just the amino acid composition, and it also incorporates the evolutionary information from the amino acid sequence. Compared to the typical dipeptide composition, the dimension of the proposed CTM feature is smaller significantly.

#### The proposed feature extraction method of absolute entropy correlation analysis based on PSSM (AECA-PSSM)

The element *P*_*ij*_ in PSSM indicates the related probability of the amino acid in the *i*th position evolves into a particular amino acid type. Therefore, each column in a PSSM can be regarded as a probability distribution. And for a PSSM, there are 20 columns in total, so that we can obtain 20 probability distributions in a PSSM. To further extract protein sequence information from the position-specific scoring matrix (PSSM), the absolute entropy correlation analysis method (AECA-PSSM) is proposed for the expression of proteins. The AECA-PSSM is a method based on the relative entropy method, and it is used to analyze the pairwise relationship between each two columns of PSSM.

Relative entropy [53], also known as Kullback-Leibler divergence (KL divergence or KLD) or information divergence, is an asymmetric method which is used to measure the difference between two probability distributions. So it is desirable to naturally analyze information in PSSM utilizing the relative entropy based methods. The relative entropy (KL divergence) between two different probability distributions can be described as follows:

7 $$ \begin{aligned} D_{KL}(P||Q)&=\sum_{i=1}^{N}P(i)log\left(\frac{1}{Q(i)}\right)-\sum_{i=1}^{N}P(i)log\left(\frac{1}{P(i)}\right)\\ &=\sum_{i=1}^{N}P(i)log\left(\frac{P(i)}{Q(i)}\right) \end{aligned}  $$

According to Gibbs inequality, KL divergence is always non-negative. When it equals to 0, it means that the two distributions are the same. And it is obvious that *D*_*KL*_(*P*||*Q*)≠*D*_*KL*_(*Q*||*P*), so the KL divergence doesn’t absolutely reflect the distance between two variables. If we directly use the KL divergence to analyze the information embedded in PSSM, we need a 20×19=380 dimensional vector because of the asymmetry of KLD. In order to make the relationship between two variables to satisfy the commutative law, the absolute entropy is calculated by:

8 $$ \begin{aligned} D(P,Q)&=\frac{1}{2}(D_{KL}(P||Q)+D_{KL}(P||Q))\\ &=\frac{1}{2}\sum_{i=1}^{N}(P(i)-Q(i))log\left(\frac{P(i)}{Q(i)}\right) \end{aligned}  $$

The absolute entropy is also always non-negative and zero also represents that the two distributions are the same ones. Through absolute entropy, the difference between two signals can be uniquely determined.

For the PSSM which we have stated to consider as 20 probability distributions, the absolute entropy correlation analysis is employed between each two probability distributions. Finally, for a protein sequence, a 20×19/2=190 dimensional feature vector is established through AECA-PSSM.

As the above, for each protein sequence, it can be described as a 230-dimensional feature vector by fusing the 40-dimensional consensus sequence-based transition matrix (CTM) feature and 190-dimensional absolute entropy correlation analysis (AECA-PSSM) feature.

### Linear discriminant analysis for dimensionality reduction of the proposed features

Though more information can be learned by combining multiple features, it also results in more irrelevant and redundant information which imposes a burden on the classifier. Dimensionality reduction is an effective way to resolve this problem. Hence, linear discriminant analysis (LDA) [54], a supervised dimensionality reduction method is employed to reduce the dimension of the proposed features and eliminate the noise.

LDA [55] is one of the most popular dimensionality reduction methods. Given a data set with *n* protein samples $\left \{x_{i},y_{i} \right \}_{i=1}^{n}$, where *x*_*i*_∈*R*^*d*^ is the feature vector of the protein sample and *y*_*i*_∈{1,2,⋯,*C*} is the corresponding class label of the sample. Let *π*_*c*_ be the subset corresponding to protein samples with label *c* and contain *n*_*c*_ data points, $\sum _{c=1}^{C}n_{c}=n$. We write $X=\left [x_{1},x_{2},\dots,x_{n}\right ]$. The within-class scatter matrix *S*^(*ω*)^ and the between-class scatter matrix *S*^(*b*)^ are separately defined as follows.

9 $$\begin{array}{@{}rcl@{}} S^{(\omega)}=\sum_{c=1}^{C}\sum_{x_{i}\in\pi_{c}}(x_{_{i}}-m_{c})(x_{i}-m_{c})^{T} \end{array} $$

10 $$\begin{array}{@{}rcl@{}} S^{(b)}=\sum_{c=1}^{C}n_{c}(m_{c}-m)(m_{c}-m)^{T} \end{array} $$

where $m_{c}=\frac {1}{n_{c}}\sum _{x_{i}\in \pi _{c}}x_{i}$ is the class mean of *π*_*c*_, and $m=\frac {1}{n}\sum (x_{i})$ is the global mean of all samples. The optimization criteria of LDA is to seek a linear transformation that maps the samples in the high dimensional space to a lower dimensional space, such that the between-class scatter is maximized and the within-class scatter is minimized. Therefore, the optimization objective of LDA is as follows:

11 $$\begin{array}{@{}rcl@{}} W^{*}=\mathop{\arg\max}_{W}\left[tr\left(\frac{W^{T}S^{(b)}W}{W^{T}S^{(\omega)}W}\right)\right]  \end{array} $$

where *W*^∗^ is the projection matrix. And the objective can be solved by generalized eigenvalue problem *S*^(*b*)^*W*=*λS*^(*ω*)^*W*. And the optimal projection matrix *W*^∗^ can be constructed by taking the eigenvectors of (*S*^(*ω*)^)^−1^*S*^(*b*)^ consistent with the *p*,(*p*<*C*) largest eigenvalues.

The projection of LDA can be obtained through Eq. (12):

12 $$ Q=(W^{*})^{T}X   $$

Through LDA, the original high-dimensional feature is projected into a lower-dimensional space and the complexity of the classifier is decreased.

### Support vector machine (SVM)

Support vector machine (SVM) [56] is a well-known supervised algorithm proposed by Vapnik. The core principle of SVM is to find a classification hyperplane to maximize the distance between positive and negative samples. SVM is built on statistical learning theory. More precisely, it is the approximate realization of minimum structural risk. When faced with samples which are linearly inseparable in low-dimensional space, SVM utilizes the kernel function to render them linearly separable in high-dimensional space. In this work, we choose a radial basis function (RBF) to solve a nonlinear problem. The equation of RBF is defined as:

13 $$\begin{array}{@{}rcl@{}} K(x,x_{i})=exp\left(-\gamma||x-x_{i}||^{2}\right),\gamma >0 \end{array} $$

LIBSVM toolbox [57] is used in this work to train the classification model. SVM is originally designed for two-class classification problems, and when it comes to the multi-class classification problem, such as the protein subcellular location, it is necessary for us to build appropriate multi-class classifiers. LIBSVM toolbox uses one-versus-one (OVO) strategy to solve multi-class classification problems. The specific method is to construct an SVM classifier between any two kinds of samples, so that if there are *k* categories we will get *k*(*k*−1)/2 SVM classifiers. When categorizing a sample unlabeled, the category that gets the most votes is the final class of the unknown sample.

### Model validation and performance evaluation

In our experiment, the jackknife test is used to evaluate the effectiveness of the classifier [4]. Jackknife test can get a unique result and it is deemed to be the most objective and reasonable. For a given dataset, the jackknife test needs to test every sample in the dataset. The principle of the jackknife test is to select one sample from the dataset as an independent test sample, and use the remaining samples for training until all the samples in the dataset have been tested. For example, as for the CL317 dataset which contains 317 apoptoisis proteins, each protein sequence will be treated as a test sequence, and the remaining 316 sequences will be used to train the classification model. After all 317 sequences were tested, the result is achieved.

Furthermore, to evaluate the performance of the model more comprehensively, six evaluation metrics including sensitivity (Sen), specificity (Spe), accuracy (Acc), Matthews correlation coefficient (MCC), F-measure (F) and the overall accuracy (OA) are used in this paper, which can be calculated as follows:

14 $$\begin{array}{@{}rcl@{}} Recall_{i}\ or\ Sen_{i}=\frac{TP_{i}}{TP_{i}+FN_{i}} \end{array} $$

15 $$\begin{array}{@{}rcl@{}} Spe_{i}=\frac{TN_{i}}{TN_{i}+FP_{i}} \end{array} $$

16 $$ \begin{aligned} &MCC_{i}\\ &=\frac{TP_{i}\times TN_{i}-FP_{i} \times FN_{i}}{\sqrt{\left(TP_{i}+FP_{i}\right)\left(TP_{i}+FN_{i}\right)\left(TN_{i}+FP_{i}\right)\left(TN_{i}+FN_{i}\right)}} \end{aligned}  $$

17 $$\begin{array}{@{}rcl@{}} {Acc}_{i}=\frac{TP_{i}+{TN}_{i}}{TP_{i}+{TN}_{i}+{FP}_{i}+{FN}_{i}} \end{array} $$

18 $$\begin{array}{@{}rcl@{}} {Precision}_{i}=\frac{TP_{i}}{TN_{i}+{FP}_{i}} \end{array} $$

19 $$\begin{array}{@{}rcl@{}} F_{i}=2\times \frac{Recall_{i}\times {Precision}_{i}}{Recall_{i}+{Precision}_{i}} \end{array} $$

20 $$\begin{array}{@{}rcl@{}} OA=\frac{\sum_{i=1}^{c}{TP}_{i}}{\sum_{i=1}^{c}({TP}_{i}+{FN}_{i})} \end{array} $$

where *TP*_*i*_, *FN*_*i*_, *TN*_*i*_, *FP*_*i*_ represent the true positive rate, false negative rate, true negative rate and false positive rate of category *i* respectively.

### The detail of the CTM-AECA-PSSM-LDA subcellular location prediction method

**Fig. 5:**
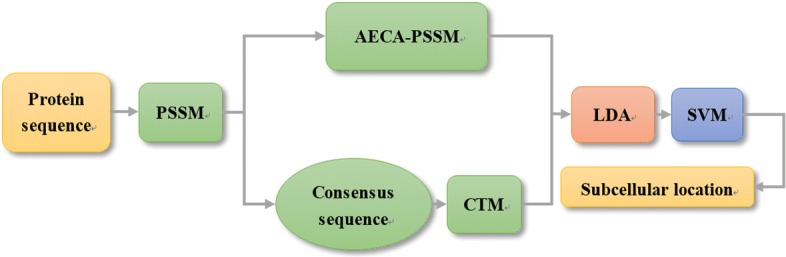
Pipeline of the proposed CTM-AECA-PSSM-LDA method for predicting apoptosis proteins subcellular location

The detail of the model which used to predict the subcellular location of apoptosis proteins is as follows. The pipline of this proposed method is shown in Fig. 5. For convenience, the proposed method is called CTM-AECA-PSSM-LDA.

Step 1: Input the protein samples in CL317 dataset and ZW225 dataset, respectively. Using CTM a 40-dimensional feature vector is generated and a 190-dimensional feature vector is extracted by AECA-PSSM. By combining these two different features, a 230-dimensional feature vector is established.

Step 2: Using LDA dimensionality reduction method to reduce the redundancy of the 230-dimensional feature vector.

Step 3: Employing SVM to identify the subcellular locations of apoptosis proteins.

## Data Availability

All the datasets and source code can be downloaded from https://github.com/LEI-SHUN/PSL.

## Abbreviations

AECA: Absolute entropy correlation analysis
Acc: Accuracy
CTM: Consensus sequence-based transition matrix
ELM: Extreme learning machine
F: F-measure
KNN: K-nearest neighbors
LDA: Linear discriminant analysis
LR: Logistic regression
MCC: Matthews correlation coefficient
OVO: One-versus-one
OA: Overall accuracy
PSSM: Position-specific scoring matrix
SVM: Support vector machine
Sen: Sensitivity
Spe: Specificity
